# Legal Sexes and Genders Should Be Plural—If They Are Justifiable at All

**DOI:** 10.1007/s10508-025-03361-6

**Published:** 2025-11-15

**Authors:** Anna Katharina Mangold, Christoph Rehmann-Sutter

**Affiliations:** 1 Europa-Universität Flensburg, Auf Dem Campus 1, E24943 Flensburg, Germany; 2 Institute for History of Medicine and Science Studies, University of Lübeck, Königstraße 42, E23552 Lübeck, Germany

## Introduction

The two questions we address —“How many sexes are there?” and “How many genders are there?”—depend on disciplinary context and the discursive vantage point. They require different answers because both “sex” and “gender” mean different things in different fields. There is an irreducible plurality of conceptualizations. Palm (2025) distinguishes at least five: (1) biology, (2) medicine/psychology, (3) the social sciences, (4) cultural studies, and (5) legal/normative studies. In biology, sex relates to reproduction and evolution, while gender has little purchase. Medicine views sex through the lens of developmental disorders or reproductive dysfunction; psychology supports patients therapeutically, e.g., in gender transition. The social sciences investigate practices of “doing gender” and the social roles attached to sex and gender. Cultural studies explore the meanings of sex and gender and how those meanings change historically, including what is taken to be “biological sex.”

Legal and normative studies, in turn, approach sex and gender primarily as regimes that establish a social order and as categories that lead to differential treatment of people. These disciplinary perspectives interact in complex ways, but none subsumes the others. A comprehensive account of the complex phenomenon of sex and gender must at least acknowledge these different ideas of sex/gender.

In our contribution, we consider the two questions solely from a legal point of view, within a conception of law suitable for liberal democracies. We argue that respect for human rights—including the right not to be discriminated against on grounds of sex/gender—requires liberal democracies to legally accept individual’s own perceptions of their sex/gender in law. A legally prescribed, mandatory sex/gender registration allocated by third parties (such as physicians or parents) cannot be justified under the constitutional requirement of proportionality. We develop this argument by asking: (1) How are legal categories constructed?; (2) What is law’s role in constructing gender/sex?; (3) How is the sex/gender binary stabilized?; and (4) How does the human rights principle of proportionality bear on legal gender/sex recognition?

## Categorizations in Law

Although many countries tend to conceive sex and gender in binary terms, a growing number of jurisdictions, Germany among them, have been allowing for more than two gender options in identity documents and public registers (Quinan & Hunt, 2023). Once a legal–normative category such as sex/gender with subcategories is established, it becomes necessary to define the category and its subcategories. Subcategories must be separated clearly and rigorously. In law, each concrete case can be subsumed under only one subcategory; it cannot be allocated to more than one; otherwise, the distinction is rendered legally superfluous. If “sex” (or “gender”—the distinction is irrelevant at this point) is legally established, it must be determined how many, and which, subcategories should exist. The traditional subcategories “female” and “male” must also be clearly distinguished.

Definitions may be created within the legal system for solely legal purposes (e.g., “female is someone who declares herself to be female”). Alternatively, the law can refer to concepts of “sex” or “gender” originating in other disciplines, thereby enshrining those external definitions (e.g., “female in the legal sense is what medicine or biology defines as female”). Even where new subcategories such as “diverse,” “non-binary,” or “without assignment” are added, the need for legal definition remains, and the law must specify what these mean in legal terms. In this sense, legal categories are constructed: Definition creates the category and fixes how many subcategories there are. This is true not only for sex/gender but for other legal categories of persons (e.g., race, religion, political belief; Mangold, 2021, pp. 305–346). Considered as a “social practice,” law uses definitions as a way of ordering and regulating the world, so that it can be decided which concrete case falls under which subcategory, and, consequently, which rules apply. Because law must decide which rules apply in specific situations, it needs clear demarcations; definitions may therefore vary across domains (e.g., schools, prisons, healthcare). Most legal systems, however, use one central definition: A person is assigned a gender/sex subcategory—typically at birth—and registered as such. Since the newborn cannot yet express themselves, third parties (parents, midwife, doctor, nurse, registrar) make the assignment.

## Invisibilizing the Social Constructedness of Legal Categories

Legal regulation and practice powerfully establish and maintain social categories, including the crucial sex/gender categories. This ordering through law exercises considerable power and is open to critique from the standpoint of justice. The staple defense is to render human agency—and its arbitrariness—invisible. Invisibility is achieved through a three-step argumentative sequence of claiming naturalness, normality, and neutrality of the installed distinctions. We describe this process as an argumentative three-step: The categorizing distinctions between humans are first alleged to be “natural” and secondly to be “normal,” while the law is, thirdly, completely “neutral” in this regard (Mangold, 2021, p. 313).

First, distinctions between groups of persons are alleged to be “natural”—already existing and simply found in nature (e.g., the distinctions between black and white people, between men and women, or between persons with and without disability). Secondly, the claim of naturalness is flanked by a claim of normality: The distinction is unquestionable, has always been there, and is universally accepted; hence, it is utterly “normal.” It has been claimed that, for instance, all civilized people would distinguish between blacks and whites, that everywhere on earth women and men are different, or that individuals with or without impairment do not have the same capabilities. Thirdly, law is cast as entirely “neutral” in regard to this categorizing: It merely reacts to these preexisting (“natural” and “normal”) distinctions rather than constructing them; differential treatment is therefore justified.

This third claim of legal neutrality conceals law’s actually very active role in creating categories and subcategories—for example, the “one-drop rule” in racial categorization, the mandatory assignment of a newborn to one of two sexes, or the legal distinction between disease and disability, which is still frequently used by courts. Not only are intra-category variants portrayed as reproducing “natural” distinctions (such as the different variants within the category of sex/gender); implicitly, a judgment is also cast on what counts as “normal” because it can be neatly classified. Doubtful cases are resolved by decision. The prevailing presumption is that the number of variants is finite, although human individuality displays effectively infinite variation—as is especially obvious in the case of racialized traits and skin colors.

The threefold claim that a certain human categorization was “natural” and “normal” and the law entirely “neutral” in this regard, hides the active role of law and legal practice in the political and societal processes of assigning humans to different categorically defined groups. Foucault (1977) described the institutional and discursive power of law and legal practice as “normalizing power”—a power that produces the normal and the abnormal.

## Strategies to Stabilize the Binary

Drawing on Foucault, psychoanalysis, and Kristeva, Butler (1990) conceptualized abjection as the process by which a discrete subject is constructed through exclusion. The abject is expelled as a misfit, and is then “discharged as excrement, literally rendered ‘Other’” (Butler, 1990, p. 133). Abjects are constructed as the not-us. By this exclusion, the accepted order is consolidated. Political philosopher Ludwig (2011) stressed the simultaneity of establishment and abjection: The abject has a constitutive role for the consolidated order. In a two-sex/gender system, casting the abject out stabilizes the binary. In consequence, the abject is denied legal existence; affected individuals must shelter under ill-fitting established categories and live a legal life incognito.

Abjection is only one stabilizing strategy. Another is what we call the binary appropriation of the non-binary: explaining the misfit (e.g., intersex) as an interweaving of the two recognized sexes. If successful, this maneuver eliminates the tension between categories and misfit. The existence of intersex people or of those who live a non-binary gender identity is then deemed no refutation of the hegemonic two-sex/gender order: As long as their existence can be redescribed as a superposition or combination of two-sex/gender components, the order can be defended against recalcitrant reality. A botanical analogy illustrates the point: Many plants (e.g., roses) bear both male and female sexual organs in the same flower; others (e.g., the hazel) bear them at different sites or times on the same individual. A monoecious plant does not represent a third sex. Monoecy is a sex system (The Tree of Sex Consortium et al., 2014)—a way of distributing reproductive organs across individuals—that still organizes two reproductive sexes.

Stock (2021), a defender of the two-sex view, argues that most cases within “Disorders/Differences of Sex Development” (DSD, or “intersex”) can easily be accommodated within philosophically defensible binary models—the gamete, chromosome, or “cluster” (homeostatic property cluster) accounts. Even so, Stock concedes that in some rare cases, intersex conditions cannot be explained by the binary. Stock retracts the universality of the claim of “sex binary”: “Properly understood, the ‘sex binary’ requires only that the vast majority of people fall into one category or the other. And on the three understandings of sex offered above, they do” (p. 59). Most people have either XX or XY chromosomes and only a small minority with certain types of differences of sex development (those with complete androgen insensibility syndrome) have female body characteristics and an XY genotype; a vast majority of people can be categorized according to differently sized gametes etc. As a legal defense, however, this is a non-sequitur: Justice must accommodate everyone, especially minorities who would otherwise be disregarded.

In Germany, where since 2018 a third legal variant “diverse” has been recognized, that subcategory arguably needs to be interpreted as diverse in its own terms (Rehmann-Sutter, 2024). Jurisdictions with a third option must clarify whether it is (1) a third gender category in its own right, (2) a diversely compiled umbrella, or (3) a formulaic compromise without ontological implications. Without clarification, “diverse” risks becoming the abject of the binary—a dumping ground for all who unsettle it—thereby shoring up heteronormative binarism.

## The Human Rights Requirement of Proportionality

As a matter of fact, people’s sex/gender self-perception (1) may change across a lifetime; (2) one’s own gender perception is not only determined by the body; (3) a human rights perspective implies that legal registration must not depend on surgery or psychiatric assessments; and (4) there are gender/sex self-perceptions beyond female and male that law must reflect. These human rights requirements have been reflected both in national constitutional law and regional human rights regimes such as the European Convention on Human Rights (Mangold, in press). Rationally reconstructed within a liberal-democratic understanding of law and state (Habermas, 1996, p. 287), the baseline is that, in liberal democracies, individuals decide how they want to live and how they define themselves. Illiberal, right-wing governments, by contrast, often force people into predetermined categories (Dietze & Roth, 2020; Gunda-Werner-Institute, 2022). This can currently be observed when it comes to legal sex/gender assignment. If a liberal state interferes with individuals’ freedom to determine their own sex/gender through legal requirements and their implementation, it must provide a valid justification. This is because any interference with a human or civil right must be justified under human rights and constitutional law and is subject to judicial review—the rule-of-law guarantee of individual freedom vis-à-vis state authority (Bingham, 2007).

The central criterion is referred to as proportionality (Möller, 2021). The proportionality test asks whether: (1) The regulation or measure pursues a legitimate purpose permitted by the constitution and human rights; (2) it is suitable for achieving that purpose; (3) it is necessary—there being no equally suitable but less intrusive means of achieving the intended purpose; and (4) the interference with human rights is proportionate to the aim pursued.

It is unclear what legitimate purpose general legal registration of a person’s gender/sex serves. The principle of proportionality requires to raise the question: Why register sex/gender at birth when the newborn cannot yet express themselves? Why register sex/gender at all? Why so few options, especially in legal systems that still presume a binary? A justification is needed, mandated by human rights. Why use a single sex/gender entry across disparate areas of law, even where sex/gender is irrelevant (e.g., road traffic law, which all people must comply with regardless of their sex/gender)? We argue that liberal states cannot provide convincing answers to these questions. Where no justification is available, general legal registration of gender/sex lacks a legitimate purpose. In a liberal state, there is no overarching reason to categorize all people by their sex/gender. Hence, categorizing people before they can express themselves cannot generally be justified.

A less intrusive (and thus potentially proportionate) approach would allow individuals to choose their sex/gender subcategory upon reaching a certain age (e.g., majority). However, in a legal perspective, assignment cannot simply be an act of power but needs to respect human rights of the individual, particularly the human right to sex/gender autonomy. Therefore, self-perception of one’s own sex/gender legally must serve as basis of legal gender/sex recognition. And for this, two subcategories do not suffice: The law must recognize more than just “male” and “female.”

Nor is a catch-all such as “diverse” (as it exists in Germany) adequate; who says “I am diverse”? In a human rights perspective, legal terms have to correspond to how people describe themselves, pointing to an open-ended number of legally recognizable sex/gender subcategories. Finally, where sex/gender is irrelevant (e.g., road traffic), no gender/sex category should be recorded.

By contrast, targeted, area-specific regulation (e.g., anti-discrimination law) is justifiable to address concrete patterns of discrimination and provide remedies: Equal pay for equal work applies to all; yet, historically, women have been paid less and men hold disproportionate power. Measures to promote women can therefore remain permissible and desirable on human rights grounds. Historical inequalities must be legally challengeable—but only in specific domains; a one-size-fits-all registration regime overshoots the mark.

## Conclusion

Only area-specific regulations and measures that pursue clearly identifiable legitimate purposes are justifiable in light of the human right to be legally recognized in one’s own sex/gender perception. The principle of proportionality requires states to allow individuals to determine their own sex/gender and, as far as possible, to refrain from interference through general legal registration. Where the category of sex/gender is regulated sector-specifically, legal recognition must extend beyond male and female. A collective label such as “diverse,” however well-intended, remains discriminatory if it does not reflect non-binary individuals’ self-perception.
